# Automated macular hole staging on optical coherence tomography using an optimized ResNet-18 framework

**DOI:** 10.3389/fcell.2026.1886103

**Published:** 2026-07-02

**Authors:** Zou Jianjun, Xu Tao, Yang Bo, Zeng Fujiang, Huang Xiaoming, Shi Saiqing, Liu Hui, Zhang Rongzhu, Dan Xiaofen, Wu Tong

**Affiliations:** 1 Aier Eye Hospital, Jinan University, Guangzhou, China; 2 Chengdu Aier Eye Hospital, Chengdu, China; 3 School of Electronics and Information Engineering, Sichuan University, Chengdu, China; 4 Aier Sichuan Eye Hospital, Chengdu, China

**Keywords:** artificial intelligence, deep learning, macular hole, ophthalmic imaging, optical coherence tomography, prediction model

## Abstract

**Purpose:**

To develop and validate a deep learning model for automated macular hole (MH) staging using optical coherence tomography (OCT) images.

**Methods:**

This retrospective study included OCT-confirmed MH images collected between July 2019 and October 2024. After quality control and redundancy screening, 6,243 representative OCT images were retained. Highly similar consecutive scans were excluded through combined manual review and program-assisted similarity assessment before model development. Models were developed using standardized preprocessing and five-fold cross-validation. ResNet-based and Swin Transformer–based architectures were constructed and compared. ResNet-18 was further optimized by integrating deformable convolution (DCNv2) and efficient channel attention (ECA).

**Results:**

Swin Transformer Tiny achieved high training accuracy but showed unstable performance on the test set. In contrast, the optimized ResNet-18 demonstrated more consistent classification results across validation folds. The optimized ResNet-18 achieved accuracy, precision, recall, F1-score, specificity, and AUC values of 98.23%, 97.17%, 96.90%, 97.00%, 99.44%, and 99.76%, respectively. External validation showed consistent classification accuracy on independent data.

**Conclusion:**

The optimized ResNet-18 achieved accurate and stable MH staging using OCT images under a standardized training framework. Compared with deeper CNN and Transformer-based architectures, the proposed model demonstrated more reliable performance under limited-data conditions. These findings support the feasibility of automated MH staging using OCT images.

## 1 Introduction

Macular hole (MH) is a defect of the neurosensory retina located at the macula, involving disruption that can extend from the internal limiting membrane (ILM) down to the photoreceptor layer (Rossi et al., 2022; Govetto et al., 2020; Schumann et al., 2020; Ezra, 2021). As the disease progresses, this structural damage may result in partial- or full-thickness retinal loss. This can ultimately lead to central vision impairment, which is often irreversible if not treated in time (Rossi et al., 2022; Steel et al., 2021; Wong and Steel, 2020). In clinical practice, MH is typically staged according to the extent of retinal involvement and the size of the defect, as these factors are closely linked to both surgical decision-making and visual prognosis (Duker et al., 2013; Tanner et al., 2020; Michalewska et al., 2021). Yet, in early stages, the morphological changes can be subtle, and their interpretation often depends on clinical experience. This makes consistent and reproducible staging difficult, particularly across different clinicians or centers (Schumann et al., 2020; Maier et al., 2021; Steel, 2020).

Optical coherence tomography (OCT) is now routinely used as the primary tool for MH evaluation, largely because it allows *in vivo*, high-resolution visualization of retinal microstructure (Huang et al., 1991; Spaide et al., 2018; Fujimoto and Swanson, 2016). It provides detailed information on the vitreoretinal interface and enables tracking of disease progression, including the transition from vitreomacular traction (VMT) to full-thickness MH (Duker et al., 2013; Johnson, 2020; Duker, 2021; de Sisternes et al., 2020). This capability has improved early detection and clinical understanding of disease evolution (Spaide et al., 2018; Fujimoto and Swanson, 2016; De Fauw et al., 2018). Even so, most conventional computer-aided approaches still rely on relatively simple measurements or handcrafted features derived from OCT images (Xu et al., 2020; Venhuizen et al., 2021). These strategies tend to perform well under controlled conditions, but their performance may decline when applied to more hetero DCNv2geneous data. Variations in image quality, patient anatomy, and acquisition settings can all affect stability, limiting their usefulness in real-world clinical scenarios (Venhuizen et al., 2021; Abràmoff et al., 2018).

With the rapid development of artificial intelligence (AI), particularly deep learning (DL), there has been growing interest in applying data-driven models to retinal image analysis (Ting et al., 2019; Liu et al., 2019; Burlina et al., 2021; Esteva et al., 2019). However, achieving reliable performance for MH staging remains challenging. Limited sample size, class imbalance, and subtle inter-stage differences continue to affect model performance (Cheplygina et al., 2019; Shorten and Khoshgoftaar, 2019; Raghu et al., 2019). In this study, a transfer learning strategy is adopted to improve model robustness under limited data conditions (Pan and Yang, 2010; He et al., 2016). A structurally refined ResNet-18 is further introduced to enhance feature representation (Dai et al., 2017). The model is designed to better adapt to irregular retinal morphology by incorporating deformable convolution (Zhu et al., 2021; Wang et al., 2020), while a lightweight channel attention mechanism is used to improve feature discrimination (Woo et al., 2018; Dosovitskiy et al., 2021). To further evaluate robustness, multiple backbone networks are compared (Liu et al., 2021; Hatamizadeh et al., 2022; Topol, 2019), and the proposed method is validated on both real-world OCT data and an external multi-center dataset. The goal is not only to improve classification performance, but also to enhance the reliability and clinical applicability of MH staging.

However, reliable MH staging remains challenging because of limited sample size, class imbalance, and subtle morphological differences between adjacent stages. To address these issues, transfer learning was adopted to improve model performance under limited-data conditions. An optimized ResNet-18 architecture integrating deformable convolution and efficient channel attention was further introduced to enhance feature representation.

The present study aimed to develop and validate a deep learning framework for automated MH staging using OCT images and to evaluate its performance using external validation data.

## 2 Materials and methods

### 2.1 Study population

This retrospective study included patients diagnosed with macular hole (MH) who underwent optical coherence tomography (OCT) at Sichuan Eye Hospital between July 2019 and October 2024. After quality-control screening and redundancy removal, 6,243 representative OCT images were retained for model development. To reduce potential sampling correlation, highly similar consecutive scans from the same acquisition sequence were excluded through combined manual review and program-assisted similarity assessment before data partitioning. According to the international classification system, the dataset included 307 stage I images, 2,020 stage II images, 345 stage III images, and 3,571 stage IV images.

For external validation, an independent dataset was obtained from the Chinese Ophthalmic Database, including 343 stage I images, 19 stage II images, 250 stage III images, and 1,018 stage IV images. In addition, 419 normal OCT images were incorporated into the final test set to further evaluate model performance in distinguishing MH from normal retinal morphology. Healthy OCT images were acquired using the same clinical imaging workflow and preprocessing protocol as MH images to reduce potential acquisition-related bias.

### 2.2 Inclusion and exclusion criteria

Inclusion criteria were as follows: (1) OCT images of macular hole (MH) acquired during routine clinical examination; and (2) images with confirmed MH staging after quality-control review. To reduce potential sampling correlation, highly similar consecutive scans from the same acquisition sequence were excluded before model development.

Exclusion criteria were as follows: (1) poor image quality, severe noise, or significant imaging artifacts; (2) presence of intraocular tamponade (e.g., silicone oil or gas) or intraocular tumors; (3) incomplete visualization of the foveal region; and (4) previous macular surgery affecting retinal morphology.

### 2.3 Handling of class imbalance and experimental design

Given the imbalanced class distribution, no direct resampling strategies, such as over-sampling or under-sampling, were applied. Instead, model performance was evaluated using macro-averaged precision, recall, F1-score, specificity, and area under the ROC curve (AUC), which are less affected by class imbalance than accuracy alone.

A five-fold cross-validation strategy was adopted. In each fold, 80% of the data were used for training and 20% for testing. Class proportions were maintained approximately across folds while allowing minor variations in sample composition. Data partitioning was performed after image-level quality control and redundancy screening to reduce potential overlap between highly similar consecutive scans across folds.

### 2.4 Cross-dataset evaluation and generalization assessment

To further assess cross-dataset generalizability, the external dataset was randomly partitioned into training and testing subsets using the same proportion as the primary dataset, followed by dataset-specific retraining and evaluation to further assess cross-dataset adaptability under different data distributions. A five-fold cross-validation strategy was adopted for the primary dataset to ensure robust performance assessment. Detailed distributions of MH stages across training and testing folds are presented in Supplementary Figure S1, while complete sample allocations are summarized in Supplementary Tables S1, S2.

This retrospective study was approved by the Ethics Committee of Sichuan Eye Hospital, Chengdu. The requirement for informed consent was waived due to the retrospective nature of the study.

### 2.5 Model development

Raw OCT images showed variability in noise, aspect ratio, and clarity; therefore, a standardized preprocessing pipeline was applied. Regions of interest were automatically cropped to remove redundant background. Annotations were independently reviewed by experienced ophthalmologists and certified retinal-image graders according to standardized OCT-based staging criteria. Discrepant cases were resolved through consensus review.

**FIGURE 1:**
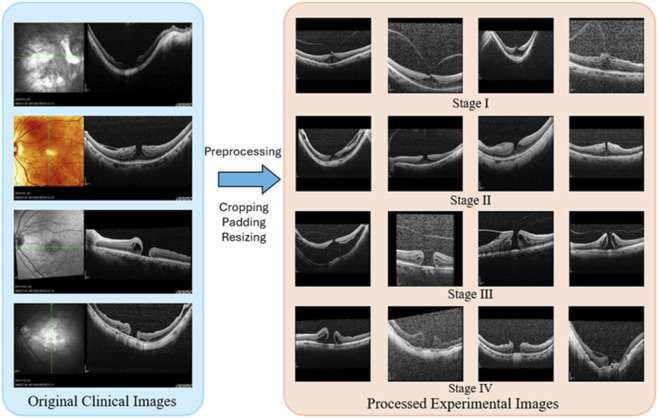
Schematic illustration of the data preprocessing pipeline.

An automated retinal region extraction pipeline was applied, including grayscale conversion, Otsu thresholding, morphological closing, and largest connected component detection. The resulting retinal regions were cropped, normalized, and resized to 224 × 224 pixels using zero-padding while maintaining the original aspect ratio. MH staging was assigned according to the four-stage Gass classification system, with de-tailed criteria summarized in Table 1. Representative preprocessing examples are presented in Figure 1.

**TABLE 1:** OCT-based staging criteria for MH.

Class	Definition	OCT characteristics
Healthy	Normal macular morphology	Intact foveal contour without retinal defect
Stage I	Impending MH	Foveal detachment without full-thickness defect
Stage II	Small full-thickness MH	Full-thickness MH < 400 μm without complete posterior vitreous detachment
Stage III	Large MH with partial vitreous attachment	Full-thickness MH ≥ 400 μm with partial vitreofoveal attachment
Stage IV	MH with complete PVD	Complete posterior vitreous detachment

μm, micrometer; MH, macular hole; PVD, posterior vitreous detachment.

Both convolutional neural network (CNN)–based and Vision Transformer (ViT)–based models were developed for comparative evaluation. CNN architectures included ResNet-18, ResNet-34, and ResNet-152 to assess the impact of network depth under limited data conditions. For transformer-based modeling, Swin Transformer Tiny (Swin-T) was employed.

**FIGURE 2:**
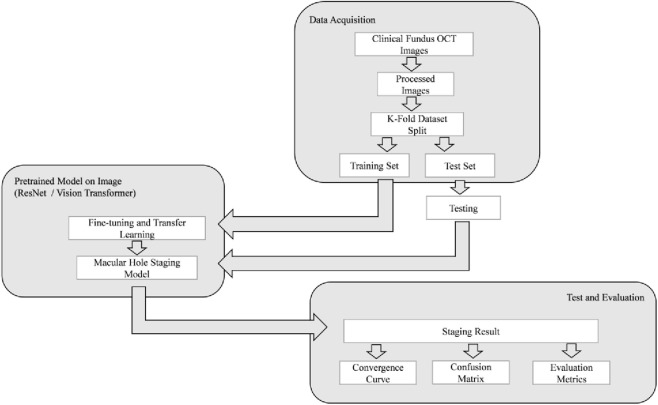
Technical workflow of the present study.

All models were implemented in PyTorch within a unified training and evaluation pipeline to ensure experimental consistency. Model performance under different training strategies was further analyzed, and the overall framework is illustrated in Figure 2.

All models were trained and tested under a unified training protocol to ensure fairness and comparability. Standard optimization strategies for classification tasks were applied. Model performance on the test set was comprehensively evaluated using accuracy, precision, recall, and F1-score.

### 2.6 Model training, evaluation, and comparative analysis

All models were trained and tested under a unified training protocol to ensure fairness and comparability. Standard optimization strategies for classification tasks were applied. Model performance on the test set was comprehensively evaluated using accuracy, precision, recall, and F1-score.

Transfer learning experiments were conducted based on ResNet-18 to examine the impact of different fine-tuning strategies on convergence behavior and staging performance. Furthermore, a comparative analysis was performed between the ResNet series and Swin Transformer Tiny (Swin-T) in terms of overall performance, class-wise recognition capability, confusion matrix patterns, and training convergence characteristics. Particular attention was given to the influence of network depth and architectural design on fine-grained staging performance, as well as the applicability and limitations of Transformer-based models in small-sample MH classification tasks. These analyses provide practical guidance for selecting appropriate model architectures and training strategies in MH staging. Performance metrics were averaged across five folds and are presented as mean values to reflect overall model stability. All models were implemented in PyTorch using identical optimization settings under the same hardware environment. Cross-entropy loss and Adam optimization were applied under a unified training framework.

### 2.7 Vision Transformer

Vision Transformers model long-range dependencies via self-attention, enabling effective global feature representation. To balance computational efficiency and modeling capacity for high-resolution medical images, Swin Transformer Tiny (Swin-T) was adopted. This architecture utilizes a shifted-window self-attention mechanism, extracting features within local windows while progressively enlarging the receptive field through a hierarchical design.

In this study, Swin-T was configured with a patch size of 4 and a window size of 7 (Swin-T-patch4-window7) to capture multi-scale structural features in MH images. Training was performed with a batch size of 128 and an initial learning rate of 0.001. Given the sensitivity of LayerNormbased architectures and the limited dataset size, the number of training epochs was increased to ensure stable convergence.

To mitigate overfitting and improve generalization, Dropout and DropPath were applied with rates of 0, 0.3, and 0.5, while other settings remained unchanged. Training loss, validation accuracy, and test accuracy were systematically analyzed to assess their impact on convergence stability and generalization performance.

Due to limited labeled medical data, all models were initialized with ImageNet-pretrained weights. For Transformer-based models, a constrained fine-tuning strategy was adopted, updating only the classification head and the final Transformer stage while freezing earlier layers to reduce trainable parameters and enhance training stability.

### 2.8 Residual networks (ResNet)

**FIGURE 3:**
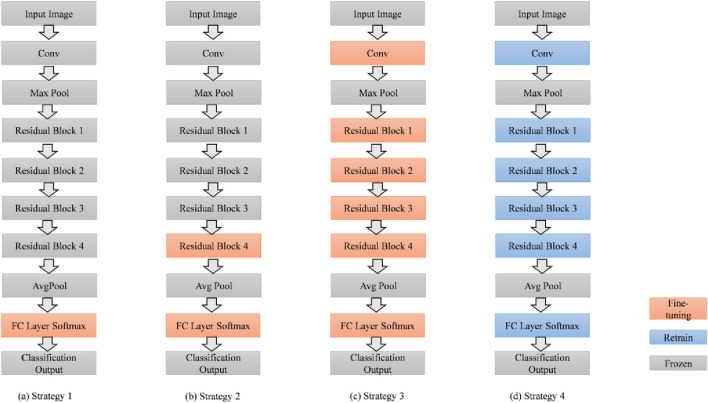
Different transfer learning strategies used in this study: **(a)** Strategy 1, training only the fully connected layer; **(b)** Strategy 2, fine-tuning Residual Block 4 and the fully connected layer; **(c)** Strategy 3, fine-tuning all residual blocks and the fully connected layer; and **(d)** Strategy 4, retraining the entire network.

To investigate the impact of transfer learning strategies on CNN performance, ResNet-18 was selected as the baseline model. Four training strategies were designed: (1) fine-tuning only the classification head; (2) fine-tuning the classification head and the fourth residual stage; (3) fine-tuning the entire network; and (4) training from scratch. By comparing convergence speed and final performance, the influence of fi-ne-tuning depth was systematically evaluated, as illustrated in Figure 3.

Experiments were conducted on Fold 1 under unified training conditions. All models were trained for 40 epochs using identical data partitioning, optimizer settings, loss function, and batch size. Training loss and classification accuracy on both training and test sets were recorded for performance evaluation.

ResNet-18, ResNet-34, and ResNet-152 were subsequently compared under the same settings (batch size = 32, learning rate = 0.001). Final performance was evaluated using five-fold cross-validation with averaged metrics calculated across all folds.

## 3 Results

### 3.1 Swin Transformer–based model

**FIGURE 4:**
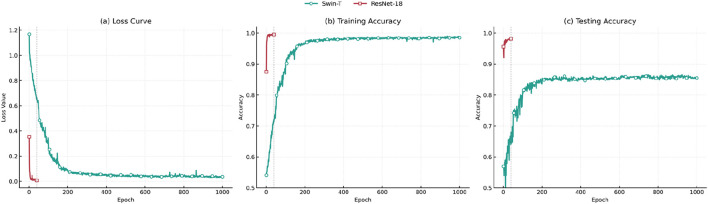
Training convergence of Swin-T on Fold 1.

Building upon the residual network experiments, a Vision Transformer–based architecture was further evaluated for MH staging. Considering the limited dataset size and moderate task complexity, the lightweight Swin Transformer Tiny (Swin-T) was selected to reduce the risk of overfitting associated with large-capacity Transformer models. All models were initialized with ImageNet-pretrained weights, and a con-strained fine-tuning strategy was adopted by updating only the classification head and the final Transformer stage to improve training stability. The convergence behavior of the Swin-T model is shown in Figure 4.

The training curves demonstrated that Swin-T converged substantially more slowly than ResNet-18, with the loss stabilizing after approximately 400 epochs. In contrast, ResNet-18 reached stable convergence within 40 epochs and achieved a low-er loss level. Although Swin-T gradually approached 99% training accuracy, the model exhibited reduced stability during optimization. More importantly, test accuracy consistently remained below 90% and fluctuated during later training stages, indicating limited generalization despite strong in-sample fitting.

**FIGURE 5:**
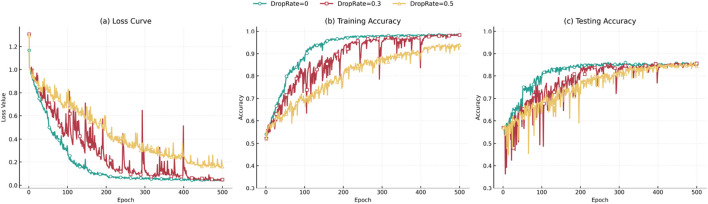
Training convergence of Swin-T on Fold 1.

To further evaluate whether overfitting could be alleviated, regularization strategies were introduced by applying Dropout and DropPath at rates of 0, 0.3, and 0.5. The corresponding convergence results are presented in Figure 5.

Increasing the regularization rate further slowed convergence and introduced more pronounced fluctuations during training. Without regularization, the model exhibited typical overfitting characterized by high training accuracy but limited test performance. Higher regularization rates resulted in slower accuracy improvement and persistent oscillations throughout training, suggesting unstable optimization. Importantly, test accuracy remained below 90% across all configurations, indicating that regularization failed to improve generalization in this task. These findings suggest that restricting model capacity reduced feature representation ability without effectively mitigating overfitting. Considering both computational cost and experimental efficiency, subsequent analyses focused on the residual network–based ResNet-18 model.

### 3.2 Residual network–based ResNet-18 model

**FIGURE 6:**
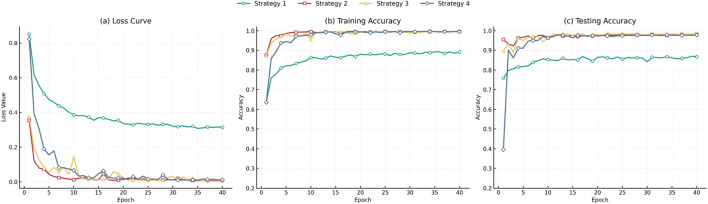
Training loss and classification accuracy curves of ResNet-18 under different transfer learning strategies on Fold 1.

Building on the previous experiments, ResNet-18 was selected as the baseline CNN model to further investigate the influence of transfer learning strategy, network depth, and architectural refinement on MH staging performance. Different fine-tuning strategies were first compared to evaluate the trade-off between convergence efficiency and classification performance. The corresponding convergence behavior and classification accuracy are presented in Figure 6.

Among the tested configurations, fine-tuning the classification head together with the fourth residual stage achieved the best balance between stability, convergence speed, and computational cost. This strategy was therefore adopted in subsequent experiments.

**FIGURE 7:**
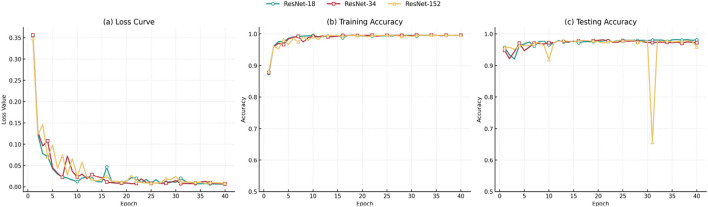
Performance of ResNet models with different depths on Fold 1.

To further investigate the effect of network depth, ResNet-18, ResNet-34, and ResNet-152 were compared under identical training settings. Model performance was assessed using training loss, training accuracy, and test accuracy curves to evaluate convergence behavior, fitting capacity, and generalization. The experimental results are summarized in Figure 7.

Analysis of the loss curves showed that ResNet-18 converged rapidly during the initial training stage and maintained stable low-loss values throughout optimization. In contrast, ResNet-34 and ResNet-152 exhibited larger fluctuations, particularly during early epochs. ResNet-152 showed pronounced oscillations in the loss curve, indicating reduced suitability for small-sample classification tasks.

All three models eventually achieved high training accuracy; however, ResNet-18 stabilized within the first 10 epochs, whereas deeper networks required longer con-vergence time and displayed greater instability. Although final test accuracy was comparable across models, ResNet-152 demonstrated noticeable declines during later training stages despite stable training performance, suggesting overfitting caused by excessive model capacity. By comparison, ResNet-18 achieved more stable convergence and reliable classification performance with substantially lower computational cost.

To further improve feature extraction, DCNv2 and ECA modules were incorporated into ResNet-18. DCNv2 enhanced adaptive spatial feature extraction, whereas ECA improved channel-wise feature representation. These modifications improved discrimination between adjacent MH stages by strengthening the network’s ability to capture subtle structural differences in OCT images. DCNv2 modules were integrated into deeper residual stages, while ECA modules were inserted after convolutional feature aggregation.

Additional evaluation was performed using an external dataset under an independent retraining setting. The improved ResNet-18 maintained stable classification performance on the external dataset, as summarized in Table 2.

**TABLE 2:** Performance evaluation of the improved ResNet-18 on the external validation set.

Metric	Accuracy	Precision	Recall	F1-score	Specificity	AUC
Result	0.9664	0.9580	0.9766	0.9663	0.9878	0.9888

Overall accuracy exceeded 96%, while the averaged recall and F1-score were ap-proximately 0.97, indicating stable and reliable differentiation across all MH stages.

**FIGURE 8:**
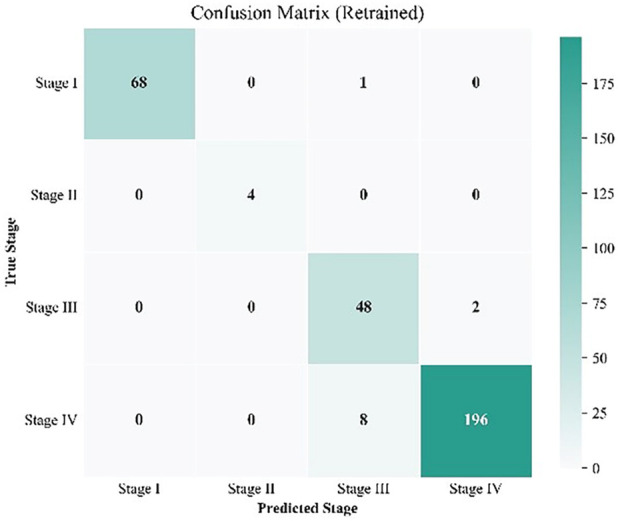
Confusion matrix of the proposed model on the external validation set.

**FIGURE 9:**
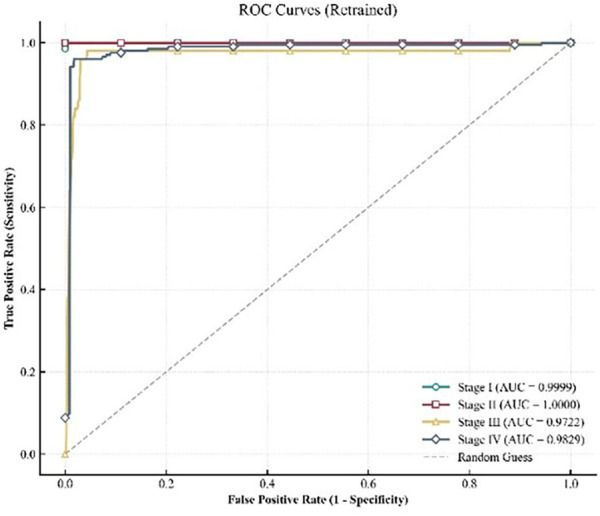
ROC curves of the proposed model on the external validation set.

Figures 8, 9 present the confusion matrix and ROC curves, respectively. Most samples were correctly classified along the diagonal, with only minor misclassification between stages III and IV. The ROC curves demonstrated strong discriminative ability, with AUC values approaching 1.0 for stages I and II and remaining above 0.97 for stages III and IV. Overall classification accuracy exceeded 96%, while the average sensitivity and F1-score were both close to 0.97, indicating stable and reliable performance of the improved model across different MH stages.

### 3.3 Integrated screening and staging performance

To evaluate model performance in distinguishing MH from normal retinal morphology, healthy OCT images were incorporated into the original stage I–IV dataset to establish a five-class classification setting. Healthy OCT images were acquired using the same imaging workflow and preprocessing protocol as MH images. The optimized ResNet-18 model was subsequently evaluated using five-fold cross-validation, and the corresponding test sample distributions are summarized in Table 3.

**TABLE 3:** Distribution of test samples of the improved ResNet-18 under five-fold cross-validation.

Fold	Healthy	Stage I	Stage II	Stage III	Stage IV	Total
1	85	70	410	58	711	1334
2	85	77	378	77	717	1334
3	85	56	404	74	715	1332
4	83	55	406	69	718	1331
5	83	49	422	67	710	1331

Because of the limited number of certain stage categories in the external dataset, particularly stage II, class-specific performance metrics should be interpreted with caution.

Classification performance across all folds is presented in Table 4. Model accuracy consistently exceeded 97%, ranging from 97.53% to 98.50%, with minimal inter-fold variation. The averaged accuracy, precision, recall, and F1-score reached 98.03%, 97.17%, 96.90%, and 97.00%, respectively, indicating that the inclusion of healthy controls did not compromise model stability or generalization performance.

**TABLE 4:** Classification performance of the improved ResNet-18 under five-fold cross-validation.

Metric	Fold 1	Fold 2	Fold 3	Fold 4	Fold 5	Mean
Accuracy	0.9843	0.9753	0.9850	0.9850	0.9820	0.9803
Precision	0.9732	0.9525	0.9879	0.9807	0.9644	0.9717
Recall	0.9709	0.9610	0.9744	0.9653	0.9735	0.9690
F1-score	0.9718	0.9565	0.9809	0.9720	0.9686	0.9700
Specificity	0.9954	0.9933	0.9942	0.9948	0.9945	0.9944
AUC	0.9979	0.9977	0.9966	0.9975	0.9985	0.9976

To further assess discriminative capability, specificity and AUC were additionally calculated. The mean specificity and AUC reached 99.44% and 99.76%, respectively, demonstrating strong classification performance in the integrated screening and staging task. Confusion matrices and ROC curves for all folds are shown in Supplementary Figures S2, S3. Most samples were correctly classified, with only minor confusion observed between adjacent advanced stages.

Overall, the improved ResNet-18 maintained high stability and generalization after inclusion of healthy controls, supporting its potential applicability in real-world clinical screening and staging scenarios.

## 4 Discussion

This study developed and validated a deep learning model for automated MH staging using OCT images. Among the evaluated architectures, the optimized ResNet-18 achieved the most stable classification performance across both internal and external validation settings. In contrast, Swin Transformer showed unstable external performance despite high training accuracy, suggesting limited suitability under the present limited-data conditions.

Accurate MH staging is important for treatment planning and prognosis evaluation. In routine clinical practice, OCT-based staging depends largely on expert interpretation and may be influenced by inter-observer variability, particularly in borderline cases. In the present study, the model provided automated classification across both healthy eyes and MH stages, which more closely reflects the sequential process of clinical screening and staging. In the extended five-class classification setting, most misclassifications occurred between adjacent MH stages, particularly among early or borderline categories. This finding is consistent with the continuous morphological progression of MH and previous reports describing the difficulty of distinguishing subtle early-stage lesions (Herath et al., 2025). Advanced-stage MH was more readily recognized because of its more pronounced structural features.

Unlike many previous medical AI studies, no over-sampling or aggressive augmentation strategies were applied in the present study. OCT images contain delicate retinal microstructures, and excessive image transformation may alter subtle anatomical characteristics or introduce unrealistic patterns (Ahmed et al., 2024; Wang et al., 2025). By preserving the original image distribution, the proposed model achieved stable classification performance while maintaining the imaging characteristics of routine clinical OCT data. To reduce the influence of class imbalance, multiple evaluation metrics, including precision, recall, specificity, F1-score, and AUC, were jointly analyzed rather than relying on accuracy alone. Consistently high AUC values and balanced confusion matrices indicated stable discrimination across different MH stages. This evaluation strategy is consistent with recent recommendations emphasizing multi-dimensional assessment in medical AI studies (Akpinar et al., 2024).

Among the evaluated architectures, CNN-based models demonstrated more stable performance than Transformer-based models. ResNet-18 achieved the best balance between classification accuracy and training stability, whereas deeper CNN variants did not provide additional benefit. Similar observations have been reported in other medical imaging studies, where increasing model complexity did not necessarily improve performance under moderate-sized datasets (Raghu et al., 2019). In contrast, Swin Transformer showed unstable external performance despite high training accuracy. Previous studies have suggested that Transformer-based architectures often require substantially larger datasets for stable optimization in medical imaging applications (Hatamizadeh et al., 2022; Toto et al., 2024). These findings support the use of relatively lightweight CNN-based architectures under limited-data conditions.

The incorporation of DCNv2 and ECA further improved classification performance. These modules may enhance adaptive spatial feature extraction and channel-wise feature representation, thereby improving discrimination between adjacent MH stages. Similar improvements have been reported in related retinal imaging studies (Zhu et al., 2021; Wang et al., 2020). However, the present study did not separately quantify the independent contribution of each module, and additional ablation analysis will be needed in future work.

Previous studies on MH-related deep learning tasks have mainly focused on multimodal classification or postoperative prediction. Xiao et al. developed a multimodal fusion network integrating imaging and textual information (Xiao et al., 2022), while Kwon et al. proposed a generative model for postoperative retinal morphology prediction (Kwon et al., 2024). In contrast, the present study focused specifically on automated staging across the full MH spectrum and included both internal and external validation.

Several limitations should be acknowledged. First, the dataset size remained moderate, particularly for certain minority-stage categories. Second, the analysis was based on two-dimensional OCT images without volumetric information. Third, although redundancy screening was performed before model development, complete patient-level separation could not be fully guaranteed in this retrospective image-based study. In addition, calibration analysis, confidence interval estimation, and formal statistical comparison between architectures were not included. Future studies incorporating multi-center datasets, volumetric OCT information, and explainable AI methods may further improve model interpretability and performance.

In conclusion, the optimized ResNet-18 achieved accurate and stable MH staging using OCT images under the present experimental setting. Compared with deeper CNN and Transformer-based architectures, the proposed model demonstrated more reliable performance under limited-data conditions. These findings support the feasibility of automated MH staging using OCT images.

## 5 Conclusion

In conclusion, this study developed and validated a deep learning model for automated MH staging using OCT images. Among the evaluated architectures, the optimized ResNet-18 achieved the most stable classification performance under the present experimental setting. The incorporation of DCNv2 and ECA further improved discrimination between adjacent MH stages, while CNN-based architectures demonstrated more stable performance than Transformer-based models under limited-data conditions.

By incorporating both healthy eyes and all MH stages, the proposed model more closely reflected the sequential process of clinical screening and staging. These findings support the feasibility of automated MH staging using OCT images and suggest the potential value of optimized CNN-based models in ophthalmic image analysis.

## Data Availability

The datasets presented in this study can be found in online repositories. The names of the repository/repositories and accession number(s) can be found in the article/Supplementary Material.
