# Supplementary material for: Automated macular hole staging on optical coherence tomography using an optimized ResNet-18 framework
Source: Front Cell Dev Biol. 2026 Jul 2;14:1886103. doi: 10.3389/fcell.2026.1886103 (PMC13373410; doi:10.3389/fcell.2026.1886103)
Supplement: Supplementary file 1 [file DataSheet1.docx]

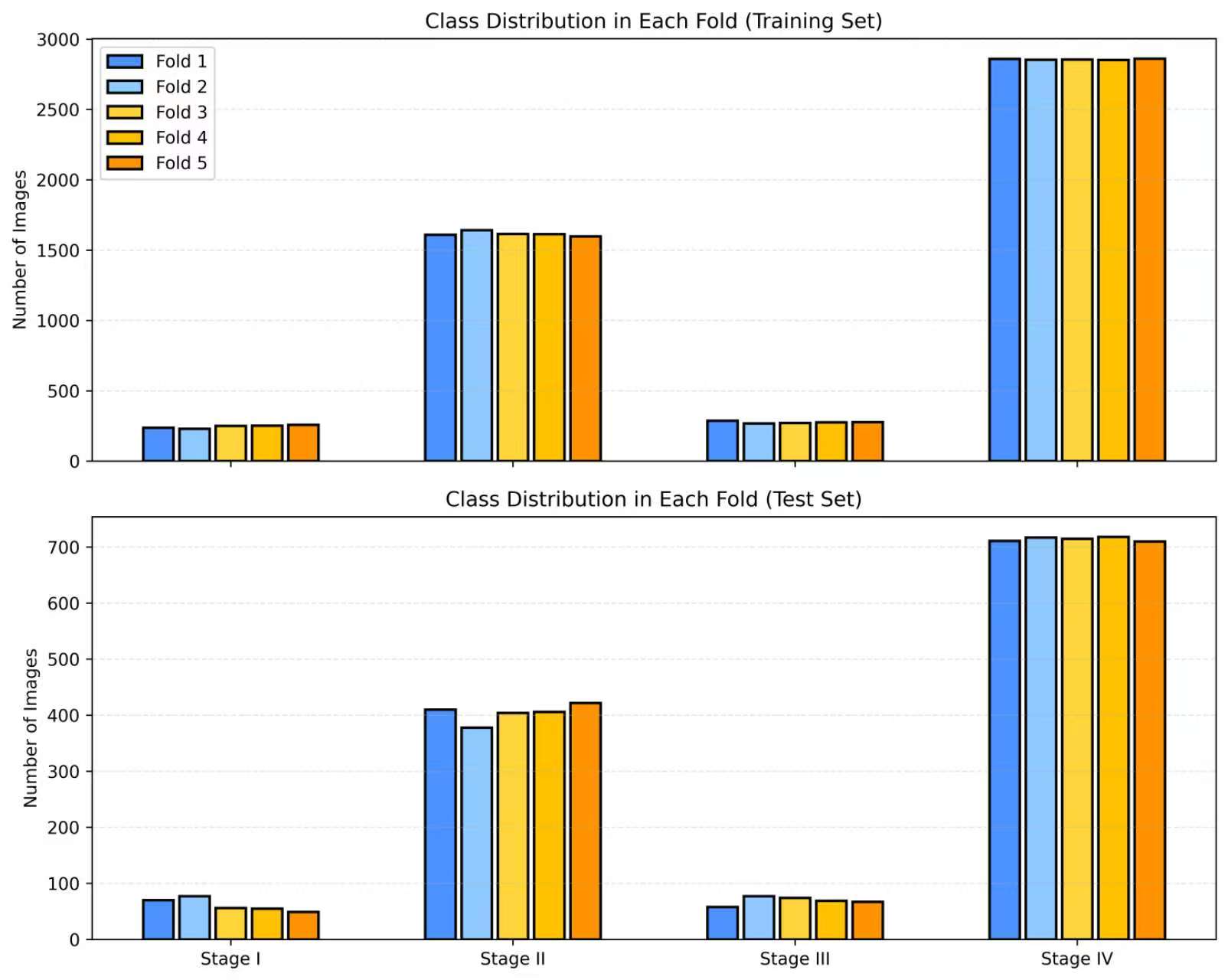
**Figure S1.** Distribution of MH stages across training and testing folds during five-fold cross-validation.

**
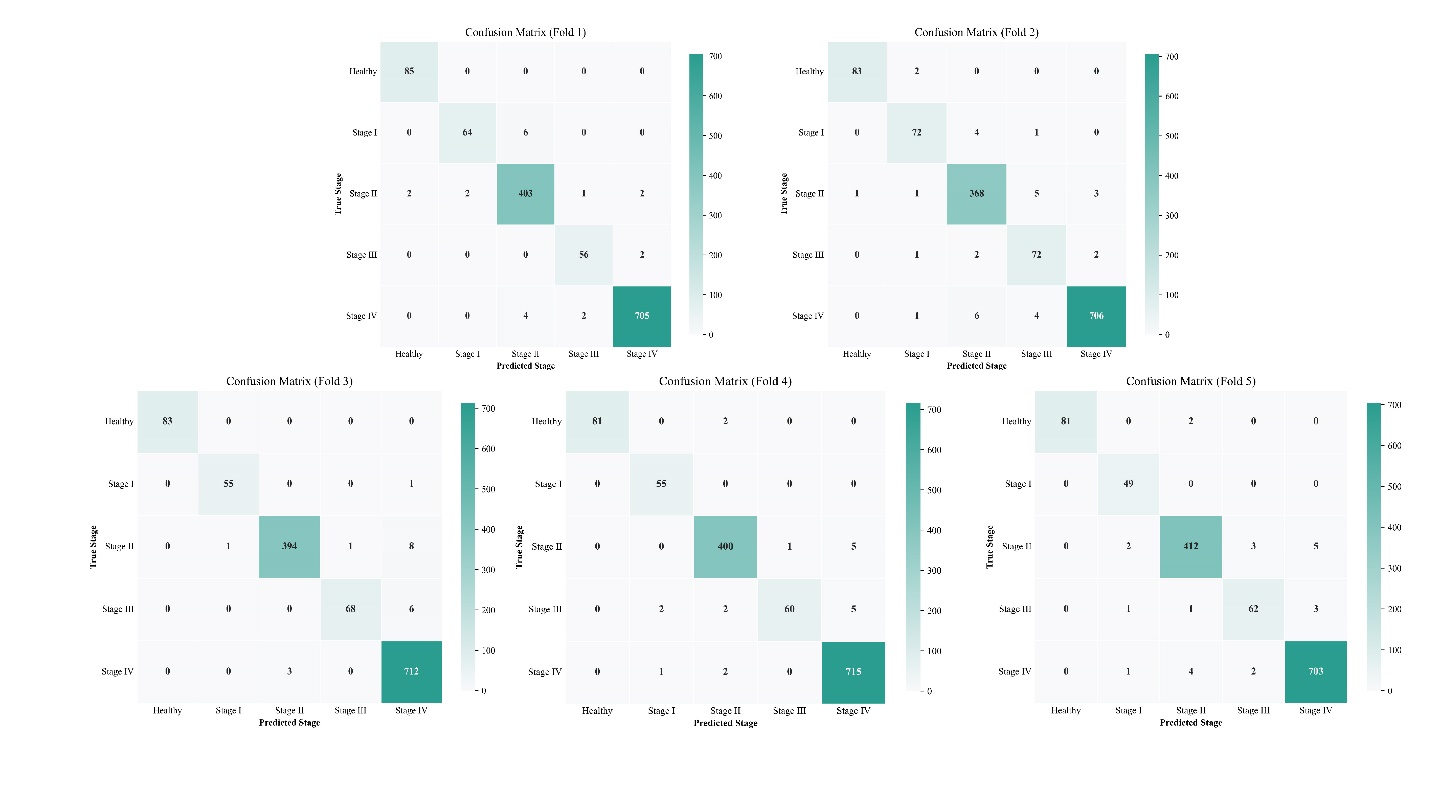
**

**Figure S2.** Confusion matrices of five subsets including healthy controls.

**
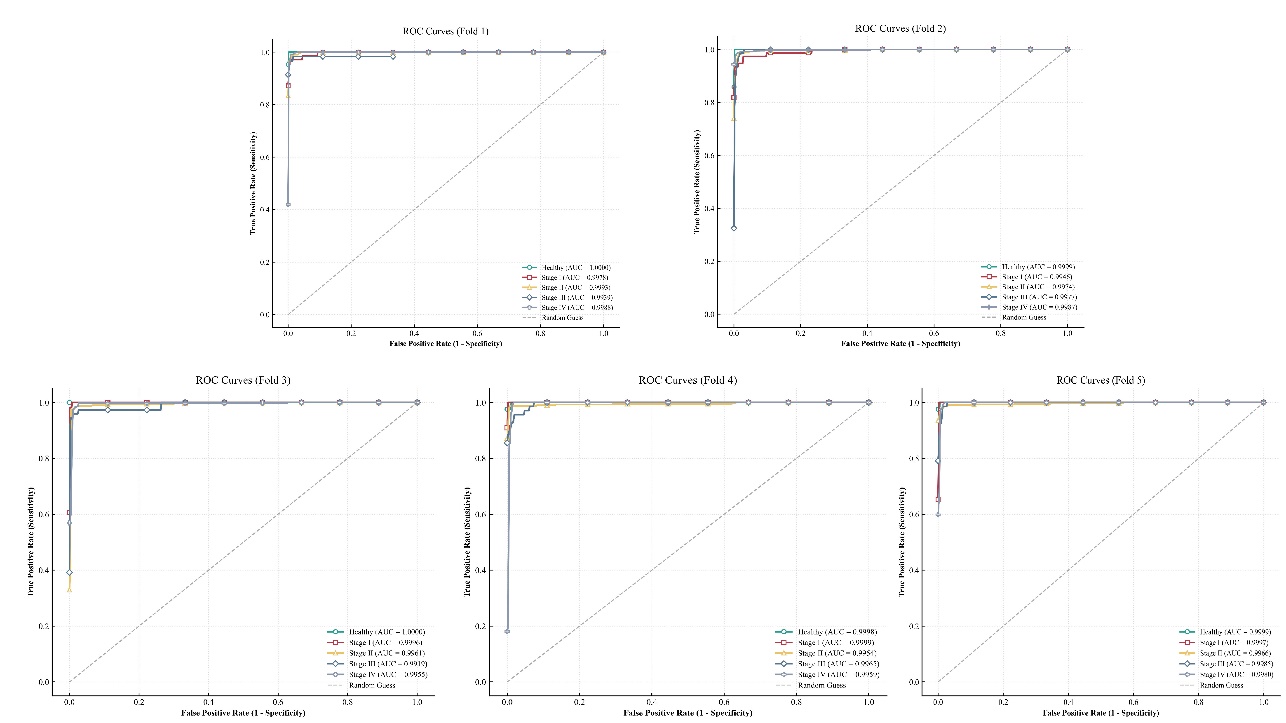
**

**Figure S3.** ROC curves for five subsets including healthy controls.

**Table S1.** Distribution of Samples Across Five-Fold Cross-Validation.

| **Fold** | **Subset** | **Stage I** | **Stage II** | **Stage III** | **Stage IV** | **Total** |
| --- | --- | --- | --- | --- | --- | --- |
| 1 | Training | 237 | 1610 | 287 | 2860 | 4994 |
|  | Testing | 70 | 410 | 58 | 711 | 1249 |
| 2 | Training | 230 | 1642 | 268 | 2854 | 4994 |
|  | Testing | 77 | 378 | 77 | 717 | 1249 |
| 3 | Training | 251 | 1616 | 271 | 2856 | 4994 |
|  | Testing | 56 | 404 | 74 | 715 | 1249 |
| 4 | Training | 252 | 1614 | 276 | 2853 | 4995 |
|  | Testing | 55 | 406 | 69 | 718 | 1248 |
| 5 | Training | 258 | 1598 | 278 | 2861 | 4995 |
|  | Testing | 49 | 422 | 67 | 710 | 1248 |

**Note:** Stage I–IV represent different stages of macular hole. The dataset was split using five-fold cross-validation, with each fold containing a training and testing subset.

**Table S2.** Distribution of the dataset by stage.

| **Subset** | **Stage I** | **Stage II** | **Stage III** | **Stage IV** | **Total** |
| --- | --- | --- | --- | --- | --- |
| Training | 274 | 15 | 200 | 814 | 1303 |
| Testing | 69 | 4 | 50 | 204 | 327 |

**Note:** Stage I–IV denote different stages of macular hole. The dataset is divided into training and testing subsets.
